# Negative enrichment by immunomagnetic nanobeads for unbiased characterization of circulating tumor cells from peripheral blood of cancer patients

**DOI:** 10.1186/1479-5876-9-70

**Published:** 2011-05-19

**Authors:** Zhian Liu, Alberto Fusi, Eva Klopocki, Alexander Schmittel, Ingeborg Tinhofer, Anika Nonnenmacher, Ulrich Keilholz

**Affiliations:** 1 Department of Hematology and Medical Oncology, Charité, Berlin, Germany; 2 Institute for Medical Genetics, Charité, Berlin, Germany; 3 Translational Radiobiology and Radiooncology Research Laboratory, Department of Radiotherapy, Charité, Berlin, Germany

## Abstract

**Background:**

A limitation of positive selection strategies to enrich for circulating tumor cells (CTCs) is that there might be CTCs with insufficient expression of the surface target marker which may be missed by the procedure. We optimized a method for enrichment, subsequent detection and characterization of CTCs based on depletion of the leukocyte fraction.

**Methods:**

The 2-step protocol was developed for processing 20 mL blood and based on red blood cell lysis followed by leukocyte depletion. The remaining material was stained with the epithelial markers EpCAM and cytokeratin (CK) 7/8 or for the melanoma marker HMW-MAA/MCSP. CTCs were detected by flow cytometry. CTCs enriched from blood of patients with carcinoma were defined as EpCAM+CK+CD45-. CTCs enriched from blood of patients with melanoma were defined as MCSP+CD45-. One-hundred-sixteen consecutive blood samples from 70 patients with metastatic carcinomas (n = 48) or metastatic melanoma (n = 22) were analyzed.

**Results:**

CTCs were detected in 47 of 84 blood samples (56%) drawn from carcinoma patients, and in 17 of 32 samples (53%) from melanoma patients. CD45-EpCAM-CK+ was detected in pleural effusion specimens, as well as in peripheral blood samples of patients with NSCLC. EpCAM-CK+ cells have been successfully cultured and passaged longer than six months suggesting their neoplastic origin. This was confirmed by CGH. By defining CTCs in carcinoma patients as CD45-CK+ and/or EpCAM+, the detection rate increased to 73% (61/84).

**Conclusion:**

Enriching CTCs using CD45 depletion allowed for detection of epithelial cancer cells not displaying the classical phenotype. This potentially leads to a more accurate estimation of the number of CTCs. If detection of CTCs without a classical epithelial phenotype has clinical relevance need to be determined.

## Background

In a variety of neoplastic diseases, the investigation of circulating tumor cells (CTCs) and minimal residual disease in bone marrow have recently gained considerable attention. CTCs can be detected in a proportion of patients with various carcinomas, and their presence has been correlated to clinical outcome [1-4]. Their detection has been recently included as a new item in the international tumor staging systems [5,6].

Detection of CTCs using reverse transcriptase PCR (RT-PCR) in peripheral blood has been explored by many investigators, including our own group over the past 15 years. Recent technical improvements have introduced the possibility of bead-based isolation of rare tumor cells from peripheral blood samples [7-10]. The currently available techniques of magnetic-bead-based enrichment and subsequent phenotyping analysis of rare tumor cells from clinical samples facilitate their detailed characterization. Furthermore, these techniques can be employed under sterile conditions, allowing the enrichment of a small tumor cell population from peripheral blood, which may be grown in culture for functional investigations in order to elucidate their biology.

The most common approaches for detection of CTCs consist of positive immunomagnetic enrichment based on frequently expressed surface markers, followed by flow cytometry or immunocytochemical analysis for visualization and quantification. Immunomagnetic separation was successful on clinical samples, and superior to the standard Ficoll density centrifugation technique [11]. The CellSearch System (Veridex LLC) is a semi-automated technique largely used in CTC isolation and detection in several cancer entities. It has been approved by the FDA (Food and Drug Administration) for detection of CTCs in advanced breast, colon and prostate cancer [12-14].

As the most commonly used techniques are based on positive selection of CTCs, only CTCs with sufficient expression of the selection marker may be enriched. Therefore, CTCs with low or absent expression of the target protein are generally excluded. This potential limitation may specifically affect the analysis of CTCs derived from tumors with down-regulation of surface epithelial markers such as EpCAM. For this reason, depletion of the leukocyte fraction (CD45 depletion) for enrichment of CTCs would be an alternative to positive enrichment strategies.

Recently, our group has developed a reliable method that allows separation of CTCs from patients with melanoma and their subsequent characterization [15]. The method is based on red blood cell lysis to remove erythrocytes, followed by depletion of leukocytes using a magnetic bead separation technique, and subsequent phenotypic characterization by multicolor flow cytometry.

In this study, the negative enrichment strategy using depletion of CD45+ leukocytes was compared to positive enrichment of EpCAM+ cells. The negative enrichment protocol was applied for detection of CTCs in a cohort of patients with metastatic carcinomas or melanoma.

## Materials and methods

### Comparison of three different enrichment methods

#### Spiking Experiments

The human colon adenocarcinoma cell line SW620 expressing EpCAM (>99%) and CK (>99%) was cultured in RPMI 1640 containing 4 mmol/L glutamine and supplemented with 20% fetal calf serum (FCS) at 37°C in air containing 5% CO_2_. Cells were harvested by incubation with phosphate-buffered saline (PBS) containing 5 mM ethylenediaminetetraacetic acid (EDTA) for 10 min at 37°C. After washing with PBS containing 2 mM EDTA, cells were counted, and their viability was assessed by trypan blue dye exclusion. One hundred SW620 cells were spiked into 5 mL blood from healthy volunteers, and enriched by means of three different methods in order to test their performance. Assays were repeated three times.

To assess the specificity of the methods (CD45 depletion and EpCAM-positive enrichment) a total of 15 blood samples from healthy volunteers were also analyzed.

#### CD45 Depletion Method

Red blood cell lysis buffer (154 mM NH4Cl, 10 mM KHCO_3 _and 0.1 mM EDTA in deionized water) was used to remove erythrocytes, and the remaining cells were washed with PBS containing 0.5% bovine serum albumin (BSA) and 2 mM EDTA. Cells were resuspended in this buffer at a concentration of 1 × 10^8 ^cells/mL. The enrichment of tumor cells by CD45 depletion of the leukocyte fraction was performed using the Human CD45 Depletion Kit (EasySep^®^, Stem Cells Technologies, Inc., Vancouver, BC, Canada) following the manufacturers' instructions with only minor modifications. In particular, magnets and buffers were kept at 4°C before use, and beads were added at a 2.2:1 ratio to the CD45 Depletion Cocktail (EasySep^®^, Stem Cells Technologies). The CD45-depleted fraction was split into two, and stained with either a cocktail of specific antibodies, or the corresponding isotypic controls purchased from the same manufacturer. All antibody batches were titrated to determine their optimal concentration. Cells were surface stained with a cocktail containing the antibodies EpCAM (clone EBA-1, BD Biosciences, San José, CA, USA) and CD45 (clone TU116, BD Biosciences) by incubating the cells in 100 μL PBS for 10' at 4°C. Cells were then washed with PBS, and fixed with 1% formaldehyde for 20' at 4°C before permeabilization for intracellular staining. To permeabilize the cells, pellet was resuspended in 2 mL of a sterile solution containing 0.1% saponin, 0.05% NaN_3 _in Hanks' Balanced Salt Solution (SAP buffer). Cells were centrifuged at 200 × g for 5 minutes; supernatant decanted ensuring that approximately 200 μL of SAP buffer remained in the tube. Cells were subsequently stained with antibodies specific for cytokeratin (CK) 7 and 8 (clone CAM 5.2, BD Biosciences), and incubated for 20 minutes in the dark at 4°C.

#### Positive Selection Method (EpCAM positive enrichment)

After the erythrocytes have been removed by red blood cells lysis buffer, the cells were resuspended in PBS + 0.5% BSA + 2 mM EDTA at a concentration of 1 × 10^8 ^cells/mL, and stained by EpCAM-Fitc (BD Biosciences) for 15 min at 4°C. Cells were then enriched by means of EasySep^® ^Fitc Positive Selection Kit (Stem Cells Technologies) according to manufacturer's instruction. Cells labeled with EpCAM Fitc-conjugated antibody are then labeled with dextran coated magnetic nanoparticles using bispecific tetrameric antibody complexes. The complexes recognize both dextran and the Fitc-molecule of the EpCAM antibody. The cell suspension was brought to a total volume of 2.5 mL, and the tube was placed into the previously cooled magnet. After 5 minutes, the supernatant was discarded, and the cells remaining in the tube were collected. Magnetic enrichment was repeated twice. Cell suspension was finally split in two fractions and stained with CD45 (BD Biosciences) and CK 7 and 8 (BD Biosciences), or the corresponding isotypic control antibodies as described above.

#### Combination of Negative and Positive Enrichment

To address whether the combination of both methods may improve results in terms of recovery and purity, a combined protocol consisting of CD45 depletion followed by EpCAM-positive selection was applied.

### Calibration Curve

The cell line SW620 was employed to obtain a calibration curve for the CD45-depletion method according to the following procedure: cells were harvested by incubating with PBS containing 5 mM EDTA for 10 min at 37°C. After washing with PBS containing 2 mM EDTA, cells were counted, and their viability was assessed by trypan blue dye exclusion. Zero, 10, 50, 100, 500 SW620 cells were respectively spiked in 5 mL blood from healthy volunteers. After CD45 depletion, the remaining cells were stained as previously described, and subsequently analysed by flow cytometry. The assay was repeated 3 times to validate the reproducibility of the method.

### Patients' Specimens

#### Samples Collection

The investigation was approved by the Ethics Committee at Charité. After informed consent, peripheral blood samples anticoagulated with heparin were collected from patients with metastatic carcinomas or melanoma receiving systemic chemotherapy at our Department. Blood was drawn after discarding the first 2 mL, to avoid potential skin cell contamination from venipuncture, and then processed within 1 hour after sampling.

Pleural effusion specimens from patients with non-small cell lung cancer (NSCLC, n = 2) and squamous cell carcinoma of the head and neck region (SCCHN, n = 1), and ascitic fluid from patients with gastric (n = 2), colon cancer (n = 1) and ovarian cancer (n = 1) were collected.

#### Flow Cytometry

After enrichment for CTCs, cells were analyzed using a FACSCanto II system (BD Biosciences). The number of CTCs in 10 mL blood was calculated by means of counting beads (BD Biosciences). Epithelial CTCs were defined as EpCAM+, CK7/8+, and CD45-. Melanoma CTCs were defined as being positive for melanoma-associated chondroitin sulfate proteoglycan (HMW-MAA/MCSP, Miltenyi Biotec Inc., Auburn CA, USA), and negative for CD45. Data were analyzed with the use of FlowJo 7.2.5 software (Tree Star, Ashland, OR, USA).

### Statistics

Data analysis was carried out with Stata statistical packages (Stata corporation, College station, TX, USA). Mann-Whitney test was used to compare the difference between the medians of CTCs of epithelial cancer patients and melanoma patients. *P *< 0.05 was considered significantly different.

## Results

### Performance of three different enrichment methods

Purity and recovery of spiked SW620 cells were compared for the three different enrichment methods: positive selection, CD45 depletion and the combination of both (CD45 depletion followed by positive enrichment for EpCAM). One hundred SW620 cells were spiked into three tubes containing 5 mL blood drawn from healthy volunteers each, and processed according to the protocols described above. The assays were repeated 3 times. Results are shown in Table 1. The recovery after CD45 depletion alone was higher than the one obtained by EpCAM-positive selection or by the combination of both (58% vs. 25% vs. 22.5%, respectively). We therefore chose to use CD45 depletion for CTC analysis in cancer patients. Three times the number of leukocytes was removed by positive selection and by the combination of the both methods, in comparison to sole CD45 depletion. However, the purity remained in the order of 1% with all three methods.

**Table 1:** Enrichment performance of the three different methods after spiking 100 SW620 cells in 5 mL peripheral blood (all assays were repeated 3 times).

Method	Total number of leukocytes		Recovery		Purity
	
	Before enrichment	After enrichment	Average (%)	Range (%)	Average (%)
CD45 depletion	3 × 10^7^	6.0 × 10^3^	58	50-66	0.97%
Positive enrichment	3 × 10^7^	2.0 × 10^3^	25	24-26	1.25%
CD45 depletion + positive enrichment	3 × 10^7^	1.5 × 10^3^	22.5	20-25	1.50%

To evaluate the specificity of the methods presence of EpCAM+CK+CD45-, EpCAM+CK-CD45- and EpCAM-CK+CD45- cells were analyzed in 15 peripheral blood samples from healthy volunteers. No EpCAM and CK double-positive cells could be detected in any of the samples. We did not observe EpCAM+CK- cells (0/15), whereas we observed the presence of EpCAM-CK+ cells in 2 samples (2/15 = 13%) when cells were enriched by CD45 depletion. The median number of CK+ cells was 2/10 mL blood with an overall false positive rate <0.5 cell/10 mL blood. After EpCAM-positive enrichment, we did not observe EpCAM+CK- cells (0/15), whereas we observed presence of EpCAM-CK+ cells in 1 sample (1/15 = 7%).

### Linearity of CTC enrichment by CD45 depletion

**Figure 1:**
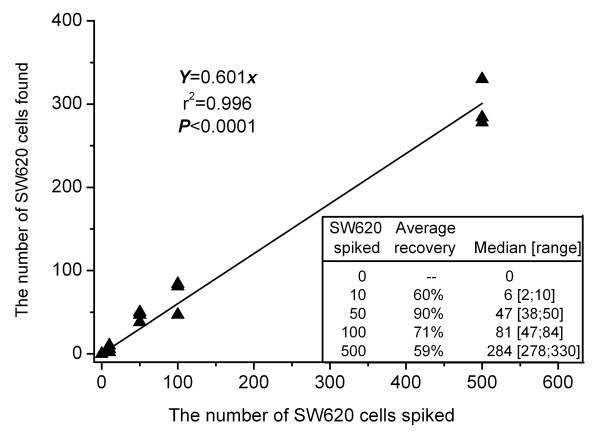
**Calibration curve obtained by CD45 depletion in spiking experiments (*n *= 3) using SW620 cells at different dilutions**.

The linear regression equation obtained by enriching spiked SW620 cells by means of CD45 depletion was calculated according to the median recovery obtained in three different experiments (Figure 1). The recovery ranged from 57% to 94% (median 69%). CD45 depletion decreased leukocyte numbers from 3 × 10^7 ^to 4~6 × 10^4 ^cells which, depending on the number of tumor cells spiked, corresponded to relative CTC level, ranging from 0.1% to 1% of all events. The enrichment process was linear for the tested concentrations (R^2 ^= 0.996). No EpCAM and CK double-positive cells could be detected in the control samples (0 cells spiked).

### Detection of CTCs in blood samples from cancer patients

**Figure 2:**
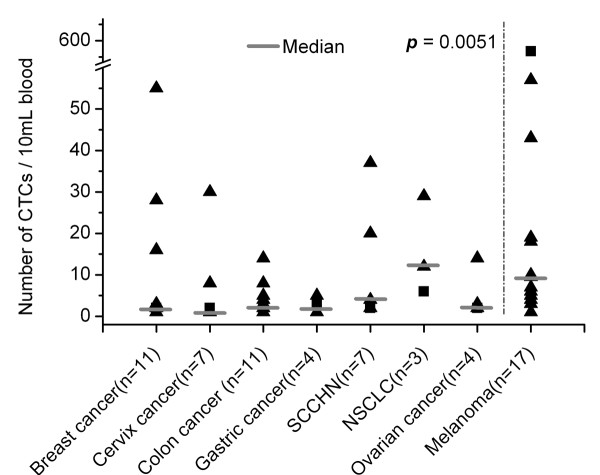
**Number of CTCs in blood samples of epithelial cancer and melanoma patients**.

CTCs were enriched by CD45 depletion and then analyzed by flow cytometry in 84 blood samples from 48 epithelial cancer patients (10 breast, 11 colon, 3 gastric, 6 ovarian, 7 cervix, 3 NSCLC and 8 SCCHN) and in 32 samples from 22 metastatic melanoma patients. Results were shown in Figure 2. CTCs could be found in 56% (47/84) of peripheral samples drawn from epithelial cancer patients, and in 53% (17/32) sample from patients with melanoma. The median number of CTCs was 3 (range: 1-55)/10 mL blood in epithelial cancer patients and 9 (range: 1-551)/10 mL blood in melanoma patients. The overall count of CTCs in melanoma patients was significantly higher than in carcinoma patients (*p *= 0.005).

Positivity detection rates were shown in Table 2. A large difference in detection rate was observed ranging from 44% in colon cancer specimens to 80% in gastric cancer samples. According to the number of patients, 33 out of 48 (69%) tested positive for CTCs. Detection rates ranged from 50% in ovarian cancer to 100% in lung cancer patients. Among 22 melanoma patients, CTCs could be found in 14 patients (64%).

**Table 2:** Detection rates of CTCs in 84 blood samples from 48 epithelial cancer patients and in 30 samples from 22 melanoma patients.

Carcinoma	Number of blood samples	Number of patients	Positivity of blood samples	Positivity of patients*
Gastric	5	3	80% (4/5)	67% (2/3)
Colon	25	11	44% (11/25)	64% (7/11)
Ovarian	8	6	50% (4/8)	50% (3/6)
Breast	21	10	52% (11/21)	60% (6/10)
Cervix	11	7	64% (7/11)	86% (6/7)
NSCLC	4	3	75% (3/4)	100% (3/3)
SCCHN	10	8	70% (7/10)	75% (6/8)
Melanoma	32	22	53% (17/32)	64% (14/22)

NOTE: * A patient was defined as positive for detection of CTCs if at least one sample resulted to be positive for presence of CTCs.

We evaluated the presence of single EpCAM or CK positive cells. In blood samples, found to be negative for presence of EpCAM+CK+CD45- cells, EpCAM-CK+CD45- cells were detected in 38% (14/37) of peripheral blood samples, and the median of the number of these cells was 6 (range: 1-43)/10 mL blood. EpCAM+CK-CD45- cells were detected in only two cases. The detection rate of EpCAM-CK+CD45- cells was significantly higher than of EpCAM+CK-CD45 cells (p = 0.001). Defining CTCs in epithelial cancer patients as CD45- CK+ and/or EpCAM+, the detection rate increased to 73% (61/84), and the median count of these cells was 8 (range: 1-105)/10 mL blood, which did not significantly differ anymore from the median count of melanoma cells (*p *= 0.418).

### Tumor cells in pleural effusion and ascites

EpCAM and CK expression levels of CD45 negative cells in pleural effusion (n = 3) or ascites (n = 4) specimens are listed in Table 3. CTCs analysis of matched peripheral blood samples is also presented for comparison.

**Table 3:** Comparison of EpCAM and cytokeratin (CK) expression profile of tumor cells in body fluids and peripheral blood samples

Cancer	Body fluids (%)			Blood (cells/10 mL)		
	
	EpCAM+ CK+	EpCAM- CK+	EpCAM+ CK-	EpCAM+ CK+	EpCAM- CK+	EpCAM+ CK-
NSCLC	<0.1%	94%	<0.1%	0	3	0
NSCLC	<0.1%	68.6%	<0.1%	0	29	0
SCCHN	69.6%	16.7%	5.8%	2	0	2
Gastric	90.2%	1.8%	2.5%	1	0	0
Gastric	<0.1%	1.3%	<0.1%	1	9	1
Colon	<0.1%	98.8%	<0.1%	15	0	4
Ovarian	<0.1%	95.7%	0.5%	2	0	0

EpCAM-CK+ cells could be found in pleural effusion specimens and in peripheral blood samples of patients with NSCLC. Cells obtained from pleural effusion have been successfully cultured (RPM1 1640 containing 20% FCS, 4 mmol/L-glutamine and 8 μg/mL tylosine) and passaged longer than 6 months suggesting their neoplastic origin. In two ascites specimens (colon cancer and ovarian cancer), CK+EpCAM- cells were detected, although EpCAM+CK+ positive cells were found in peripheral blood. Cells were successfully cultured and easily passaged for several months. The cell line derived from the patient with ovarian cancer (EpCAM-CK+) was characterized by flow cytometry for expression of different stem cells markers (additional file 1) and by Comparative Genomic Hybridization (CGH). CGH analysis revealed more than 20 genetic aberrations, including a loss of the short arm of chromosome 11 and a gain in the short arm of chromosome 19. These structural chromosomal changes confirmed the tumor origin of the cell line.

In all the other cases, a correspondence between blood and ascites, or blood and pleural effusion was observed. However, due to the small number of paired samples, a firm conclusion cannot be drawn.

## Discussion

Several recent studies showed that the phenotypic and genotypic characterization of CTCs may provide valuable information of clinical relevance [16-18]. However, unbiased CTC isolation is a crucial initial step for their subsequent characterization.

Different methods have been routinely employed for CTC enrichment and detection. The CellSearch System is a semi-automated enrichment and immunocytochemical detection system approved by the FDA, using EpCAM expression as its primary mechanism of selection of CTCs. In a cohort of metastatic breast cancer patients, an average recovery of 74.9% was obtained [19]. Enrichment by MACS columns is another technique used. This system involves tumor cells coupled with specific microbeads that are enriched by removing unlabeled cells via washing, using a column placed in a magnetic device. Recovery rates ranging 60%-80% have been reported [20]. More recently, the development of a microchip technology based on EpCAM-coated microposts capture of epithelial cancer cells allowed recoveries over 65%, and purity of over 50% [21]. All the enrichment methods mentioned above are based on the expression of surface markers on CTCs, in particular, EpCAM.

We tested three different enrichment methods (positive selection, CD45 depletion and the combination of both) in a spiking experiment model using a cell line known to be positive for EpCAM, and CK 7 and 8. We observed the highest recovery in sole CD45 depletion. In the case of EpCAM-positive selection, the recovery rate was lower compared to many other studies published. In order to evaluate if cancer cells might be lost in the non-enriched fraction, both the fractions (enriched for EpCAM-positive cells and non-enriched) were analyzed by flow cytometry. In the non-enriched fraction, we were able to find a few cells that tested CK and EpCAM positive. The mean fluorescence intensity of the EpCAM-positive cells in the non-enriched fraction resulted to be lower in comparison to the mean fluorescence of the SW620 cells and considerably lower in comparison to the fluorescence of the SW620 cells we were able to detect in the enriched fraction (data not shown). Excluding the possibility of EpCAM down-regulation after antibody binding [22], the relatively low fluorescence signal due either to inferior EpCAM surface expression, or to the weakening of the Fitc-staining (the lapse of time between staining and FACS analysis in case of positive enrichment is of at least 90 minutes compared to 25 minutes when CD45-depletion was performed) might be an explanation to the low recovery rate obtained after EpCAM-based immunoselection in accordance to the fact that the cells' recovery would increase with increasing fluorescence of the Fitc-labelled cells. Consequently, CTCs that do not express EpCAM at sufficient levels could be missed by these assays, which may limit the sensitivity, and could potentially lead to a loss of particular cell subpopulations. Indeed, heterogeneous expression of epithelial surface markers has been previously reported in different tumor entities at tissue level [23,24], as well as the loss of EpCAM expression in the case of epithelial-mesenchymal transformation [25,26].

Only a few studies applied negative enrichment for CTCs detection [27-32]. Lara et al. reported 46% average recovery rate and depletion efficiency up to 5.7 Log by enriching cells by means of a flow-through system [27]. A similar recovery rate was obtained by Zigeuner et al., who compared in spiking experiments positive selection of epithelial cells with the antiepithelial antibody BER-EP4 with CD45 depletion. Furthermore, when a single tumor cell was spiked in 30 ml, CD45 depletion revealed epithelial cells in all 14 cases, whereas positive selection in 12 of 14 cases [28]. Higher recovery rates found to be comparable to ours were obtained by Meye et al. [29] by applying CD45 autoMACS depletion. The same group also observed a significant correlation between presence of CTCs and lymph node status, and occurrence of synchronous metastases in a cohort of patients affected with renal cell carcinoma [30].

We detected CTCs after CD45 depletion in 48 epithelial cancer patients and 22 melanoma patients. The 64% of melanoma patients resulted to be positive for CTCs which is in accordance to results of a previous study from our group [33]. The median count of CTCs in melanoma patients was significantly higher than the median count of CTCs (defined as CD45-EpCAM+CK+) in carcinomas signifying that either hematogenous spread of melanoma is somehow easier, or that the definition of CTCs in carcinoma is too restrictive leading to an underestimation of CTCs when the common definition of EpCAM CK double positive is applied. However, when defining cells as EpCAM+ and CK+, our data showed similar or slightly higher detection rates compared to data reported by other authors who detected CTCs in comparable cohorts of patients (56% in metastastic breast cancer [34], 64.7% in NSCLC [35], 38% in ovarian cancer and 31% in gastric cancer [36]).

We used antibodies against CK7 and CK8 for cytokeratin detections. We chose CK7 and CK8 (always associated to expression of CK18) because they resulted to be the most expressed CKs in carcinomas along with CK19 [37]. In particular, CK8 is expressed by a variety of carcinomas. Since CK expression pattern in carcinoma is heterogeneous, addition of further anti-CK antibodies might increase the sensitivity of the detection method [38,39], but congruently the false positive rate. In our preliminary experiments, the use of CK19 as an additional antibody resulted in a higher background in healthy controls (data not shown).

We analyzed CTCs in peripheral blood and in matched pleural effusion or ascites specimens of seven patients. In five out of seven cases a correspondence of EpCAM and CK expressions was observed between CTCs, and tumor cells in ascites or pleural effusion samples. This result is consistent with the present understanding that CTCs and disseminated tumor cells released from the primary tumor tissue, i.e, with the same origin, or might re-circulate between metastatic sites [40]. However, in two cases, although EpCAM+ CK+ cells were detected in peripheral blood, CK positive cells were detected in ascites, which may be due to the fact that circulating cells with different phenotypic characteristics may specifically colonize an organ [41-43] or an anatomical space. Ascitic fluid may in this case represent a reservoir for naturally enriched, disseminated tumor cells bearing specific features as it has been shown to occur in other compartments [44]. In the two NSCLC patients, only CK- positive cells could be detected both in blood and pleural effusion. Cells obtained from pleural effusion could be passaged in culture several times, supporting the hypothesis of their neoplastic origin. An enrichment method based on EpCAM-positive selection would therefore not have been able to detect this fraction of cells. Consequently, the definition of CTCs as CD45- and EpCAM and CK double positive might be too restrictive. Loss of epithelial markers like EpCAM and CK is a common phenomenon which typically occurs in cells which undergo the epithelial-mesenchymal transition (EMT), a process that has been linked to the generation of cells with properties of stem cells, and to the ability of tumor cells to enter the circulation and seed metastases. EpCAM-CK double positive CTC might represent only a subpopulation of the whole pool of CTCs. Establishment of new assays based on EMT or stem cells markers are therefore necessary.

## Conclusion

In conclusion, CTCs enrichment based on CD45 depletion allowed the detection of epithelial cancer cells that do not show the classical epithelial phenotype potentially permitting a more likely estimation of the number of CTCs. If detection of CTCs without a classical epithelial phenotype has clinical relevance need to be determined.

## Competing interests

The authors declare that they have no competing interests.

## Authors' contributions

ZL conceived the study, collected the samples, carried out assays and measurements, performed the statistical analysis and drafted the manuscript. AF conceived the study, designed and conducted the study and drafted the manuscript. AS collected the samples and reviewed the manuscript. IT participated in design of the study. AN participated in samples collection and assays optimization. UK conceived the study and drafted the manuscript. All authors have read and approved the final manuscript.

## Supplementary Material

Additional file 1 **Expression of stem cell markers in an established ovarian carcinoma cell line**. The EpCAM-CK+ cell line derived from ascites of a patient with ovarian cancer was characterized by flow cytometry for expression of different stem cells markers. Cells resulted positive for several stem cell markers included NANOG, OCT3-4 and CD166, but negative for the most investigated marker CD133. Click here for file
