# Supplementary figures and images for: Negative enrichment by immunomagnetic nanobeads for unbiased characterization of circulating tumor cells from peripheral blood of cancer patients
Source: J Transl Med. 2011 May 19;9:70. doi: 10.1186/1479-5876-9-70 (PMC3119001; doi:10.1186/1479-5876-9-70)

## Slide 1
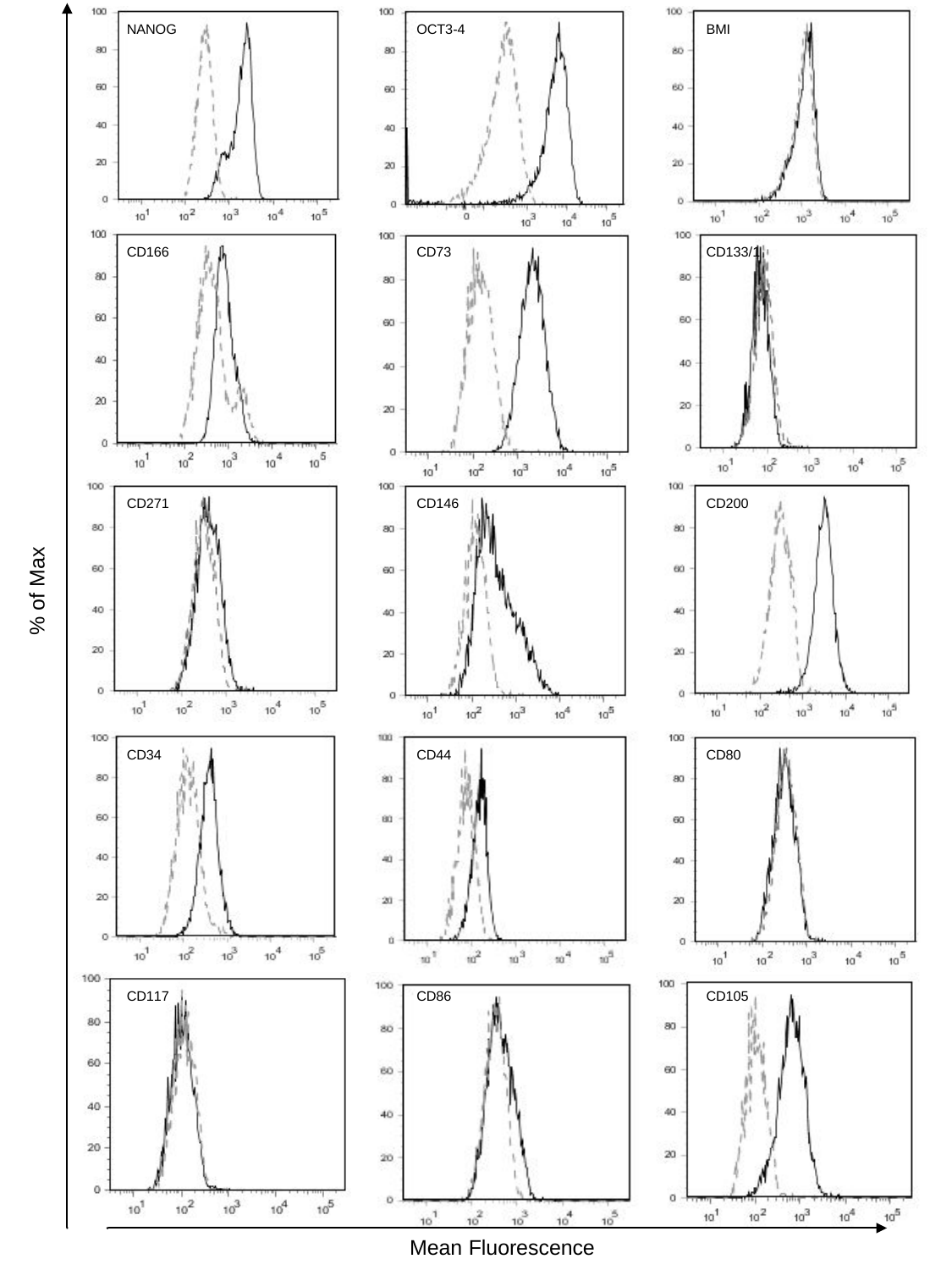

Supplement: Additional file 1 — Expression of stem cell markers in an established ovarian carcinoma cell line. The EpCAM-CK+ cell line derived from ascites of a patient with ovarian cancer was characterized by flow cytometry for expression of different stem cells markers. Cells resulted positive for several stem cell markers included NANOG, OCT3-4 and CD166, but negative for the most investigated marker CD133. [file 1479-5876-9-70-S1.PPT]
